# A Medical Inspector on the School Nurse

**Published:** 1912-04-13

**Authors:** 


### A Medical Inspector on the School Nurse.

### AN IMPORTANT NEW BOOK ON HER DUTIES.

Concurrently with the development of the system of medical inspection of school children, the school nurse has assumed a position of increasing importance. She is now not only a necessity, but a power to be reckoned with by every school doctor. Coming as she does into even more intimate contact with the parents than the school doctor himself, her influence is bound to be widely felt; and as there is no doubt that in the future school nurses will be appointed in increasing numbers, it behoves education authorities to exercise particular discretion in selecting thoroughly suitable and reliable nurses to fill these posts. At the present time there is, we fear, a tendency to cut down the salaries of school nurses to an even lower level than has been in force up till now. To expect a nurse to maintain herself entirely, to provide a bicycle, and to do efficiently work of very great importance to the community on a salary of £80 a year is none too generous treatment, but yet some authorities are now offering even less for this class of work. A glance at some of the lists of requirements drawn up by education authorities will show that the profession of school nurse is an exacting one, and that the attainments necessary for success are of no mean order.

The existence of this comparatively new and rapidly increasing class has created a demand for a text-book, or perhaps we should say rather, a guide-book, to this new profession. This want has been very efficiently met by a new publication,* written by Mr. C. L. Leipoldt, himself an experienced worker amongst school children.

As far as we know there are not in existence any special schools which devote particular attention to the training of a nurse for school work, but it is none the less a fact that her work is of a very special kind and differs enormously from hospital work. As a matter of fact, the work she has done in the wards will be of little use to her compared with the experience gained in out-patient or casualty departments, while it is obvious that a period of training spent in a children's hospital is so valuable as to be almost indispensable.

In contradistinction to purely professional attainments the school nurse should also possess some knowledge of social work and district visiting, since a large part of her work will lie in the homes of the children. Add to this already formidable list another item which is even now not infrequently demanded of candidates—namely, the possession of a certificate of the Royal Sanitary Institute—and it will readily be realised that anyone who has merely gone through a three years' course of training is still a long way from being a ready-made school nurse.

Both nurses already at work and those who have thoughts of entering this field will find Mr. Leipoldt's book packed with valuable advice and carefully compiled information. Not only are the diseases and abnormal conditions commonly met with among children of school age adequately and lucidly explained, but the hygiene of the school and of the child itself provides matter for some highly interesting and well-reasoned chapters. Mr. Leipoldt's book is the best type of nursing text-book, and the earlier chapters relating to the duties and qualities of a school nurse show a genuine appreciation not only of her difficulties but also of the great importance of this new cogwheel in our social machinery. The school medical officer will gain almost as much as the school nurse from its perusal.

* *The School Nurse: Her Duties and Responsibilities.* By C. Louis Leipoldt, F.R.C.S. London: The Scientific Press, Ltd. 1912. Price 2s. 6d.

